# Surprise, Curiosity, and Confusion Promote Knowledge Exploration: Evidence for Robust Effects of Epistemic Emotions

**DOI:** 10.3389/fpsyg.2019.02474

**Published:** 2019-11-12

**Authors:** Elisabeth Vogl, Reinhard Pekrun, Kou Murayama, Kristina Loderer, Sandra Schubert

**Affiliations:** ^1^Department of Psychology, Ludwig Maximilian University of Munich, Munich, Germany; ^2^Institute for Positive Psychology and Education, Australian Catholic University, Sydney, NSW, Australia; ^3^School of Psychology and Clinical Language Sciences, University of Reading, Reading, United Kingdom; ^4^Research Institute, Kōchi University of Technology, Kōchi, Japan; ^5^Department of Psychology, University of Augsburg, Augsburg, Germany

**Keywords:** epistemic emotion, achievement emotion, cognitive incongruity, knowledge exploration, within-person analysis, replication

## Abstract

Research has started to acknowledge the importance of emotions for complex learning and cognitive performance. However, research on epistemic emotions has only recently become more prominent. Research in educational psychology in particular has mostly focused on examining achievement emotions instead of epistemic emotions. Furthermore, only few studies have addressed functional mechanisms underlying multiple different epistemic emotions simultaneously, and only one study has systematically compared the origins and effects of epistemic emotions with other emotions relevant to knowledge generation (i.e., achievement emotions; Vogl et al., 2019). The present article aimed to replicate the findings from Vogl et al. (2019) exploring within-person interrelations, origins, and outcomes of the epistemic emotions surprise, curiosity, and confusion, and the achievement emotions pride and shame, as well as to analyze their robustness and generalizability across two different study settings (online; Study 1, *n* = 169 vs. lab; Study 2, *n* = 79). In addition, the previous findings by Vogl et al. (2019, Study 3) and the present two new studies were meta-analytically integrated to consolidate evidence on origins and outcomes of epistemic emotions. The results of the two new studies largely replicated the findings by Vogl et al. (2019). Combined with the meta-analytic results, the findings confirm distinct patterns of antecedents for epistemic vs. achievement emotions: Pride and shame were more strongly associated with the correctness of a person’s answer (i.e., accuracy), whereas surprise, curiosity, and confusion were more strongly related to incorrect responses a person was confident in (i.e., high-confidence errors) producing cognitive incongruity. Furthermore, in contrast to achievement emotions, epistemic emotions had positive effects on the exploration of knowledge. Implications for research and practice are discussed.

## Introduction

Although research has started to acknowledge the importance of emotions for complex learning and cognitive performance (see e.g., Angie et al., 2011), research on epistemic emotions, such as confusion, has only recently become of interest (e.g., D’Mello et al., 2014; Muis et al., 2015a; Arguel et al., 2019; Fayn et al., 2019; Vogl et al., 2019). In educational contexts in particular, scholars have focused predominantly on studying antecedents and performance effects of achievement emotions, such as anxiety, pride, or shame related to success and failure (Pekrun, 2018). However, epistemic emotions including surprise, curiosity, and confusion can also profoundly impact cognitive processing underlying learning and performance (Pekrun and Stephens, 2012; Muis et al., 2018a).

As such, gaining deeper understanding of the specific antecedents and effects of different emotions related to learning and knowledge generation is critically important for designing learning environments and intervention programs in a way that they foster adaptive emotions. However, the body of research for some epistemic emotions (e.g., confusion) is still sparse and ambiguous (e.g., D’Mello et al., 2014; Vogl et al., 2019). Vogl et al. (2019) is one of only a handful of extant studies addressing several epistemic emotions simultaneously to investigate their joint origins and effects (D’Mello and Graesser, 2012; Muis et al., 2015a, b, 2018b; Pekrun et al., 2017b; Trevors et al., 2017; Di Leo et al., 2019). Furthermore, to our knowledge, it is the first study that systematically compared origins and effects of these emotions with other emotions relevant to learning and knowledge generation (i.e., achievement emotions) and that used a within-person analytic approach to do so.

Given the novelty of the substantive research questions addressed by Vogl et al. (2019), as well as the lack of research addressing within-person emotional functioning, we aimed to test the robustness and the generalizability of the findings of their most extensive study (Study 3) across different settings (online Study 1 vs. lab Study 2) by conducting two direct replication studies. Direct replications are needed to test if a particular study design can reliably produce a specific empirical result (Schmidt, 2009). In addition, we used a meta-analytic approach to combine the findings of Study 3 from Vogl et al. (2019) and the results of both present studies to substantiate our knowledge on the distinct antecedents and effects of epistemic emotions. Meta-analysis increases statistical power as well as precision of estimates by pooling across studies, and thereby provides useful insights into the psychological phenomenon under study (e.g., Stanley et al., 2018). Replication and meta-analysis are both methods in line with the call for more generalizable research triggered by the “replication crisis” that has undermined the confidence in the robustness of psychological findings. Furthermore, both methods can be used to generate cumulative evidence required for developing sound, comprehensive theory that can inform practical perspectives on fostering adaptive emotions.

In contrast to the existing body of research that has predominantly focused on between-person designs (see e.g., Voelkle et al., 2014; Murayama et al., 2017), Vogl et al. (2019) used within-person analysis to examine functional relations between variables (Molenaar, 2004). Generally, empirical findings based on between-person data cannot be used to infer conclusions about the within-person relations proposed by emotion theories. While between-person approaches can be used to examine individual differences in the antecedents and effects of emotions, within-person analysis provides insight into the structures and variations of emotional states within individuals over time, in response to different task characteristics, for instance (e.g., Tsai et al., 2008; Murayama et al., 2011; Tanaka and Murayama, 2014). As such, within-person analysis is needed for gauging the validity of emotion theories that predict within-person relations between variables.

In sum, the present research seeks to provide robust evidence for a novel theoretical research area targeting antecedents and effects of an under-researched group of emotions. To do so, we used analytical methods that allowed us to derive reliable and valid conclusions about the origins and outcomes of epistemic emotions (within-person design, meta-analytic approach, and replication).

## Origins of Epistemic and Achievement Emotions

Prototypically, epistemic emotions are triggered by cognitive incongruity, which can be produced by unexpected information that contradicts prior knowledge or personal beliefs, such as high-confidence errors (Marshall and Brown, 2006). Confrontation with unexpected information can interrupt ongoing cognitive processes and shift attention to this information. Enhanced processing and the exploration of this information can be a consequence. However, the specific effects may also depend on the emotions triggered by the cognitive incongruity (Vogl et al., 2019). Surprise may be the first reaction when one is confronted with unexpected or schema-incongruous information (Stiensmeier-Pelster et al., 1995; Meyer et al., 1997; Scherer, 2009; Noordewier and Breugelmans, 2013; Noordewier et al., 2016). In addition, curiosity (Loewenstein, 1994) and confusion (Pekrun and Stephens, 2012; Silvia, 2013) may be triggered, likely following surprise (Berlyne, 1954, 1960; Loewenstein, 1994; D’Mello and Graesser, 2012; Vogl et al., 2019).

However, situations involving cognitive incongruity may also elicit achievement emotions. Persons may also feel ashamed when something they were convinced of turns out to be incorrect, or proud if they are proven correct. More specifically, achievement- related shame is triggered by internally attributed failure (e.g., lack of ability; Weiner, 1985, 2010; Pekrun, 2018), whereas achievement-related pride is triggered by internally attributed success (e.g., ability or effort; Weiner, 1985, 2010; Tangney, 1999; Tracy and Robins, 2004, 2007; Pekrun, 2018). As such, achievement emotions differ from epistemic emotions in terms of their object focus, that is, achievement outcomes (Pekrun, 2018) vs. generation of knowledge (Brun et al., 2008).

## Outcomes of Epistemic and Achievement Emotions

Epistemic emotions drive knowledge acquisition about the self and the world and are of critical importance for knowledge generation, conceptual change, and cognitive performance (Brun et al., 2008; Morton, 2010; Pekrun and Stephens, 2012; Muis et al., 2018a, b). More specifically, surprise can prompt interest, curiosity and confusion (Berlyne, 1954, 1960; Loewenstein, 1994; Renninger and Hidi, 2016; Vogl et al., 2019). It has been consistently found that curiosity promotes learning and achievement (Kang et al., 2009; von Stumm et al., 2011; Gruber et al., 2014; Marvin and Shohamy, 2016; Middlebrooks et al., 2016) as well as more specifically knowledge exploration (Berlyne, 1954, 1960; Litman et al., 2005). In contrast, effects of confusion on learning and knowledge exploration are inconsistent. Initial evidence suggests that confusion can promote learning and knowledge acquisition (Craig et al., 2004; D’Mello et al., 2014; Vogl et al., 2019) if cognitive incongruity can be resolved, as for example by engaging with the material (Brown and VanLehn, 1980; Mandler, 1990).

Similarly to confusion, findings on the effects of shame on learning and knowledge generation are inconsistent. For instance, shame has been associated with approach as well as avoidance tendencies (De Hooge et al., 2010), and it has been found to reduce intrinsic and amplify extrinsic motivation (Turner and Schallert, 2001). In contrast, pride in successful task performance has consistent positive effects on learning and achievement. Pride promotes task-oriented, extrinsic and intrinsic motivation, knowledge exploration, perseverences and effort, as well as academic achievement (Williams and DeSteno, 2008; Oades-Sese et al., 2014; Pekrun et al., 2017a, 2019).

## Epistemic Emotions and Knowledge Exploration: Findings by Vogl et al. (2019)

In three independent experimental studies, Vogl et al. (2019) used a within-person analysis to investigate the origins and relations of the epistemic emotions surprise, curiosity, and confusion, and the achievement emotions pride and shame as well as their effects on knowledge exploration. This research question is novel and relevant for educational and emotion research in several ways: first, the authors focused on epistemic emotions, one group of emotions that is highly relevant for learning and knowledge generation but under-researched; secondly, they are one of the first researchers that analyzed common antecedents and effects of epistemic emotions and compared them to origins and outcomes of one other group of emotions relevant for learning and knowledge-generation (i.e., achievement emotions). And thirdly, they used a within-person approach to disentangle within- and between-person variance which allowed them to analyze the functional relations between the selected variables (Molenaar, 2004; Murayama et al., 2017). With that, their research results can be used to validate emotion theories (e.g., control-value theory of achievement emotions; Pekrun, 2018) and can inform practice about how to create emotionally sound learning environments.

Study 1 examined surprise, curiosity, and confusion and their effects on participants’ motivation to explore the correct answer in case an incorrect answer was given. Study 2 added two prototypicall achievement emotions, namely pride and shame, to the study design and investigated the effects of all five emotions on actual exploratory behavior in case of incorrect answers. Study 3 further expanded on Studies 1 and 2 by including a broader measure of exploratory behavior by allowing participants to request up to three pieces of information per statement. In addition, they investigated the effect of the five emotions on knowledge exploration after incorrect and correct answers. Since Study 3 offered the most comprehensive picture of the relations between accuracy and confidence, emotions, and exploratory behavior, the present paper aimed to replicate Study 3.

Using a trivia task with immediate feedback, Vogl et al. (2019) found that achievement emotions were more strongly associated with accuracy (i.e., correctness of the answer), whereas epistemic emotions were more strongly related to high-confidence errors (i.e., incorrect answers an individual is confident in) generating cognitive incongruity. Moreover, as compared with pride and shame, surprise, curiosity, and confusion were more strongly and positively related to the exploration of knowledge. Specifically, surprise and curiosity were positive predictors of knowledge exploration. Confusion had positive predictive effects on knowledge exploration which were significant in Studies 1 and 3 but not in Study 2. These results were largely consistent with theoretical considerations (Loewenstein, 1994; D’Mello and Graesser, 2012; Pekrun, 2018) with the exception of the variable results of confusion. Thus, they argued that the inconsistent findings for confusion are probably due to its weak effects on knowledge exploration and concluded that in particular the effects of confusion need to be investigated further.

## Aims and Hypotheses

The first goal of this research was to directly replicate the findings of Study 3 from Vogl et al. (2019) to ensure that their study design can reliably produce the results found by Vogl et al. (2019) in different settings. Specifically, in Study 2, we collected data in a controlled lab setting instead of allowing participants to do the study online from home (Study 1). In this way, we controlled for confounding influences and distracting stimuli, such as searching the internet for the correct answer or interruptions during the study. In both studies, we used the exact same target group of participants, materials, and procedure as Vogl et al.’s (2019) in Study 3, with the exception of the different setting in Study 2 of this research. The second goal was to meta-analytically investigate the results of both present studies and the findings of Study 3 from Vogl et al. (2019) to gain a more precise understanding of the effects of the epistemic emotions and achievement emotions under study.

**FIGURE 1:**
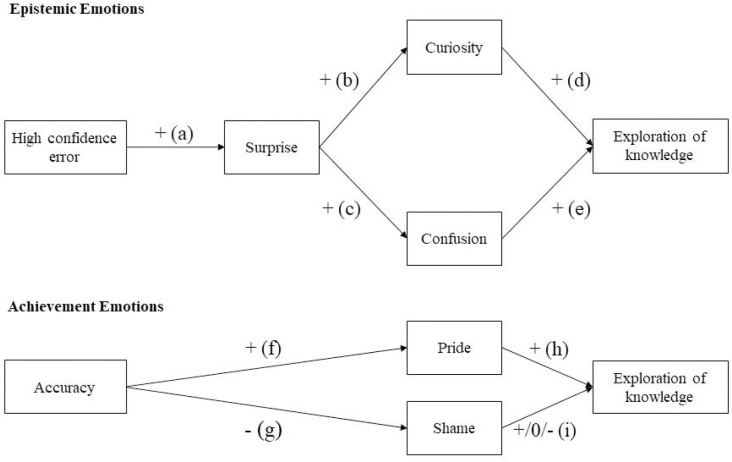
Main hypotheses.

Both studies used a trivia task with immediate feedback to trigger surprise, curiosity, confusion, shame, and pride. We expected different patterns of antecedents for epistemic emotions and achievement emotions. In more detail, we hypothesized that epistemic emotions would be primarily produced by incorrect answers a person was confident in (i.e., high-confidence errors), whereas achievement emotions would be prompted by incorrect (shame) vs. correct (pride) answers (i.e., failure vs. success). As for the outcome of the emotions, we expected surprise, curiosity, and confusion to positively predict exploratory behavior. In addition, we hypothesized that surprise would not directly predict exploratory behavior but that this effects would be mediated by curiosity and confusion. In addition, we expected pride to promote exploratory behavior after correct answers. Due to the inconsistent findings for shame, we left the direction of the effect of shame on exploratory behavior open. We tested the following hypotheses (see Figure 1) supported by Vogl et al.’s (2019, Study 3) findings anew in the present replication studies:

*Hypothesis 1:* We expected surprise, curiosity, and confusion to be positively predicted by high-confidence errors.

*Hypothesis 2:* We expected that surprise would positively predict curiosity and confusion, and that curiosity and confusion would positively predict exploratory behavior. In addition, we hypothesized that curiosity and confusion would be mediators in this effect.

*Hypothesis 3:* We expected that high-confidence errors would positively predict exploratory behavior and that epistemic emotions would be mediators in the high-confidence error-exploration relation.

*Hypothesis 4:* We expected that accuracy would positively predict pride, and negatively predict shame.

*Hypothesis 5:* We expected that pride would positively predict exploratory behavior; we did not formulate a directional hypothesis for the effect of shame on exploratory behavior.

## Study 1

Study 1 aimed to directly replicate Study 3 from Vogl et al. (2019) using the exact same target group of participants, setting (online study), materials, procedure, and methods of analysis.

### Method

#### Participants

The online study was completed by 169 (100 women, *M*_age_ = 25.53, *SD* = 7.46) from a German university. As thanks for their participation, they could win one of two €20 (US$23) gift cards.

#### Materials

Participants completed a task developed by Vogl et al. (2019) consisting of 20 items, including statements likely to trigger high-confidence errors because they target widespread errors in general knowledge to (e.g., “Ketchup is an American invention”) as well as statements with varying degrees of difficulty less likely to produce high-confidence errors (e.g., “Socrates was sentenced to die by drinking the hemlock cup”).

#### Procedure and Measures

For each of the 20 items, participants were asked to assess the accuracy of the statement and their confidence in the reply (6-point Likert scale; 1 = *very uncertain* to 6 = *very certain*). They got immediate feedback if their answer was correct (“Your answer is correct” vs. “Your answer is incorrect”) and were asked how surprised, curious, confused, ashamed, and proud they were currently feeling using a 5-point Likert scale (1 = *not at all* to 5 = *very strong)* for rating each emotion adjective (i.e., single-item versions of the Epistemic Emotion Scales; Pekrun et al., 2017b). Next, they had the opportunity to request an explanation of the statement (*“Would you like to see the explanation now?”* [*No* vs. *Yes*]) and up to two more pieces of information for each statement (*“Would you like to receive more information concerning this topic?”* [*No* vs. *Yes*]). Ethical approval was obtained by the Faculty of the first authors’ institution.

#### Data Analysis

Multilevel structural equation modeling was applied to distentangle within- and between-person relationships in the nested data (Level 1: trivia statements; Level 2: individuals) using Mplus 7.4 (type = twolevel; Muthén and Muthén, 2017). We tested two separate multilevel models:

First, we analyzed accuracy (0 = *incorrect*; 1 = *correct*), confidence, and the Accuracy × Confidence interaction as predictors of the emotions at Level 1 (Model 1) to test if high-confidence errors produce epistemic emotions (Hypothesis 1). We standardized accuracy and confidence before calculating the interaction term and centered the predictors within each individual to disentangle effects within- and between-persons (see Enders and Tofighi, 2007; Wang and Maxwell, 2015). At Level 2, variables simply correlated with each other. The model was saturated.

Secondly, we decomposed the Accuracy × Confidence interaction term to analyze its effects (Model 2). In Model 2a, we investigated the simple effects of confidence in incorrect answers (high-confidence errors) on the emotions and exploratory behavior. In Model 2b, we analyzed the same effects for correct answers. The epistemic emotions predicted exploratory behavior sequentially (Hypotheses 2 and 3): surprise predicted curiosity and confusion and both predicted exploratory behavior. 95% bootstrap confidence intervals were used to test the indirect effects. Again, variables simply correlated with each other at Level 2.

In line with recommendations by Hu and Bentler (1999), we interpreted the fit of Model 2 as good if values of the CFI were greater or equal to 0.95 and values of the RMSEA and SRMR were smaller or equal to 0.06, and as moderate if values of the CFI were between 0.90 and 0.95 and values of the RMSEA and SRMR were between 0.06 and 0.08. However, general cut-off values should be interpreted with caution (Marsh et al., 2004).

### Results

#### Preliminary Findings

Descriptive statistics and correlations are displayed in Table 1. Scores sufficiently varied for all study variables. ICCs for emotions ranged from 0.185 to 0.339, indicating that within-person variation outweighed variation at the between-person level.

**TABLE 1:** Estimated sample statistics (Means and SD) and within- and between-person correlations.

**Variable**	***M*_*cor*_**	***SD*_*cor*_**	***M*_*inc*_**	***SD*_*inc*_**	***M*_*tot*_**	***SD*_*tot*_**	**ICC**	**1**	**2**	**3**	**4**	**5**	**6**	**7**	**8**
**Study 1**															
(1) Accuracy^1^	–	–	–	–	0.56	0.08	0.028	–	0.448^∗∗^	–0.107	–0.190	–0.065	–0.063	0.071	0.008
(2) Confidence	4.01	0.62	3.84	0.53	3.94	0.58	0.165	0.052^∗∗^	–	0.262^∗^	0.241^∗^	0.231^∗^	0.371^∗∗∗^	0.218^∗^	0.034
(3) Surprise	1.63	0.50	2.81	0.72	2.13	0.56	0.185	–0.495^∗∗∗^	0.061^∗^	–	0.755^∗∗∗^	0.795^∗∗∗^	0.640^∗∗∗^	0.543^∗∗∗^	0.218^∗∗^
(4) Curiosity	2.30	0.80	3.28	0.85	2.71	0.79	0.339	–0.426^∗∗∗^	–0.120^∗∗∗^	0.611^∗∗∗^	–	0.520^∗∗∗^	0.533^∗∗∗^	0.368^∗∗∗^	0.517^∗∗∗^
(5) Confusion	1.16	0.38	2.04	0.68	1.54	0.46	0.211	–0.478^∗∗∗^	0.178^∗∗∗^	0.659^∗∗∗^	0.463^∗∗∗^	–	0.654^∗∗∗^	0.707^∗∗∗^	0.034
(6) Pride	2.12	1.01	1.11	0.35	1.66	0.60	0.311	–0.563^∗∗∗^	0.121^∗∗∗^	–0.321^∗∗∗^	–0.242^∗∗∗^	–0.348^∗∗∗^	–	0.621^∗∗∗^	0.108
(7) Shame	1.05	0.20	1.50	0.58	1.24	0.31	0.219	–0.378^∗∗∗^	0.037^∗^	0.324^∗∗∗^	0.245^∗∗∗^	0.335^∗∗∗^	–0.345^∗∗∗^	–	–0.091
(8) Exploration^2^	1.62	1.01	1.94	0.80	1.75	0.90	0.560	–0.184^∗∗∗^	–0.067^∗∗^	0.285^∗∗∗^	0.429^∗∗∗^	0.233^∗∗∗^	–0.073^∗∗^	0.119^∗∗∗^	–
**Study 2**															
(1) Accuracy^1^	–	–	–	–	0.48	0.08	0.023	–	0.209	–0.609^∗∗^	–0.399	−0.437^∗^	0.076	–0.269	0.298
(2) Confidence	3.50	0.60	3.81	0.49	3.68	0.55	0.139	–0.120^∗∗∗^	–	–0.045	0.099	–0.081	0.304^∗^	−0.241^∗^	–0.071
(3) Surprise	1.87	0.48	2.97	0.62	2.45	0.55	0.157	–0.419^∗∗∗^	0.233^∗∗∗^	–	0.680^∗∗∗^	0.761^∗∗∗^	0.231	0.563^∗∗∗^	–0.008
(4) Curiosity	2.29	0.70	3.41	0.73	2.89	0.68	0.248	–0.464	0.034	0.631^∗∗∗^	–	0.593^∗∗∗^	–0.012	0.386^∗∗∗^	0.439^∗∗∗^
(5) Confusion	1.19	0.58	2.31	0.69	1.79	0.49	0.158	–0.483^∗∗∗^	0.315^∗∗∗^	0.672^∗∗∗^	0.522^∗∗∗^	–	0.263	0.804^∗∗∗^	0.163
(6) Pride	2.37	0.28	1.06	0.20	1.68	0.51	0.198	0.628^∗∗∗^	0.049	–0.298^∗∗∗^	–0.311^∗∗∗^	–0.386^∗∗∗^	–	0.103	–0.129
(7) Shame	1.06	0.18	1.70	0.72	1.40	0.48	0.323	–0.455^∗∗∗^	0.140^∗∗∗^	0.316^∗∗∗^	0.265^∗∗∗^	0.339^∗∗∗^	–0.364^∗∗∗^	–	0.048
(8) Exploration^2^	1.44	0.87	1.81	0.77	1.63	0.79	0.497	–0.238^∗∗∗^	–0.035	0.262^∗∗∗^	0.463^∗∗∗^	0.266^∗∗∗^	–0.177^∗∗∗^	0.146^∗∗∗^	–

*ICC, Intraclass correlation coefficient. Within-person correlations are displayed below the diagonal; between-person correlations are displayed above the diagonal. ^1^Proportion of correct answers per person (range = 0.20–0.85, and 0.25–0.75 in Studies 1 and 2, respectively). ^2^Mean of the sum score of explorations (range 0–3). On average, participants answered 8.76 (SD = 2.75; Study 1) and 10.50 (SD = 2.50; Study 2) out of 20 questions incorrectly. For these incorrectly answered items, on average they explored 1.48 (SD = 1.02; Study 1) and 1.15 (SD = 0.79; Study 2) pieces of information. ^∗^*p* < 0.05, ^∗∗^*p* < 0.01, ^∗∗∗^*p* < 0.001.*

#### Origins of Epistemic and Achievement Emotions (Model 1)

Results for Model 1 are displayed in Tables 2, 3. Accuracy significantly negatively predicted surprise, curiosity, and confusion. In addition, the Accuracy × Confidence interaction was a significant negative predictor of all three epistemic emotions, confirming that high-confidence errors triggered epistemic emotions (Hypothesis 1).

**TABLE 2:** Predictors of epistemic emotions (Model 1).

	**Surprise**				**Curiosity**				**Confusion**			
			
**Predictor**	***b***	**ß**	***p***	**95% CI**	***b***	**ß**	***p***	**95% CI**	***b***	**ß**	***p***	**95% CI**
**Study 1**												
Accuracy	–0.609	–0.514	0.000	[−0.551; −0.477]	–0.478	–0.430	0.000	[−0.469; −0.391]	–0.003	–0.498	0.000	[−0.531; −0.464]
Confidence	0.120	0.094	0.000	[0.059; 0.128]	–0.113	–0.094	0.000	[−0.131; −0.057]	–0.453	0.208	0.000	[0.174;0.242]
Accuracy × Confidence	–0.602	–0.506	0.000	[−0.538; −0.473]	–0.351	–0.314	0.000	[−0.349; −0.278]	–0.204	–0.331	0.000	[−0.362; −0.299]
Confidence in incorrect answers	0.604	0.649	0.000	[0.598; 0.701]	0.262	0.345	0.000	[0.264; 0.425]	0.394	0.499	0.000	[0.434; 0.565]
Confidence in correct answers	–0.302	–0.508	0.000	[−0.570; −0.445]	–0.301	–0.405	0.000	[−0.465; −0.345]	–0.061	–0.219	0.000	[−0.304; −0.134]
Order	–0.002	–0.010	0.458	[−0.036; 0.016]	–0.004	–0.010	0.202	[−0.050; 0.011]	–0.303	–0.017	0.261	[−0.047; 0.013]
**Study 2**												
Accuracy	–0.489	–0.392	0.000	[−0.446; −0.338]	–0.522	–0.451	0.000	[−0.512; −0.391]	–0.491	–0.445	0.000	[−0.497; −0.393]
Confidence	0.203	0.163	0.000	[0.120; 0.206]	–0.015	–0.013	0.637	[−0.069; 0.042]	0.267	0.242	0.000	[0.188; 0.295]
Accuracy × Confidence	–0.661	–0.527	0.000	[−0.573; −0.482]	–0.378	–0.325	0.000	[-0.378; −0.272]	–0.359	–0.324	0.000	[−0.371; −0.278]
Confidence in incorrect answers	0.589	0.596	0.000	[0.515; 0.663]	0.267	0.315	0.000	[0.182; 0.351]	0.418	0.436	0.000	[0.337; 0.498]
Confidence in correct answers	–0.365	–0.517	0.000	[−0.437; −0.293]	–0.286	–0.340	0.000	[−0.377; −0.196]	–0.087	–0.220	0.000	[−0.139; −0.036]
Order	–0.007	–0.033	0.062	[−0.067; 0.002]	0.000	0.000	0.991	[−0.042; 0.043]	0.000	–0.001	0.947	[−0.042; 0.039]

**b*, Unstandardized path coefficient; ß, standardized path coefficient; *p, p*-value; CI, confidence interval.*

**TABLE 3:** Predictors of achievement emotions (Model 1).

	**Pride**				**Shame**			
		
**Predictor**	***b***	**ß**	***p***	**95% CI**	***b***	**ß**	***p***	**95% CI**
**Study 1**								
Accuracy	0.507	0.562	0.000	[0.519; 0.605]	–0.226	–0.384	0.000	[−0.427; −0.342]
Confidence	0.088	0.090	0.000	[0.057; 0.123]	0.037	0.059	0.001	[0.025; 0.092]
Accuracy × Confidence	0.105	0.116	0.000	[0.076; 0.155]	–0.075	–0.126	0.000	[−0.164; −0.088]
Confidence in incorrect answers	0.012	0.056	0.096	[−0.010; 0.122]	0.088	0.178	0.000	[0.096; 0.260]
Confidence in correct answers	0.100	0.179	0.000	[0.097; 0.261]	–0.009	–0.051	0.093	[−0.110; 0.008]
Order	0.000	−0.001	0.969	[−0.029; 0.028]	0.001	0.009	0.554	[−0.020; 0.037]
**Study 2**								
Accuracy	0.620	0.617	0.000	[0.563; 0.672]	–0.294	–0.433	0.000	[−0.488; −0.377]
Confidence	0.105	0.105	0.000	[0.056; 0.153]	0.058	0.085	0.000	[0.039; 0.131]
Accuracy × Confidence	0.111	0.110	0.000	[0.055; 0.165]	–0.054	–0.079	0.007	[−0.13; −0.021]
Confidence in incorrect answers	0.028	0.120	0.000	[0.004; 0.053]	0.047	0.066	0.148	[−0.013; 0.107]
Confidence in correct answers	0.211	0.232	0.000	[0.117; 0.305]	–0.032	–0.177	0.000	[−0.056; −0.008]
Order	−0.005	−0.029	0.188	[−0.073; 0.014]	0.004	0.037	0.141	[−0.012; 0.087]

**b*, Unstandardized path coefficient; ß, standardized path coefficient; *p, p*-value; CI, confidence interval.*

Furthermore, accuracy significantly positively predicted pride and significantly negatively predicted shame (Hypothesis 4). Additionally, the Accuracy × Confidence interaction term significantly positively predicted pride and significantly negatively predicted shame, indicating that participants were more proud in correct answers they were confident in and more ashamed in incorrect answers they were confident in. However, the interaction term predicted epistemic emotions more strongly than achievement emotions (β range −0.314 to −0.506 for the epistemic emotions and 0.116 and −0.126 for pride and shame; see Tables 2, 3, respectively).

#### Outcomes and Relations of Epistemic and Achievement Emotions (Model 2)

**FIGURE 2:**
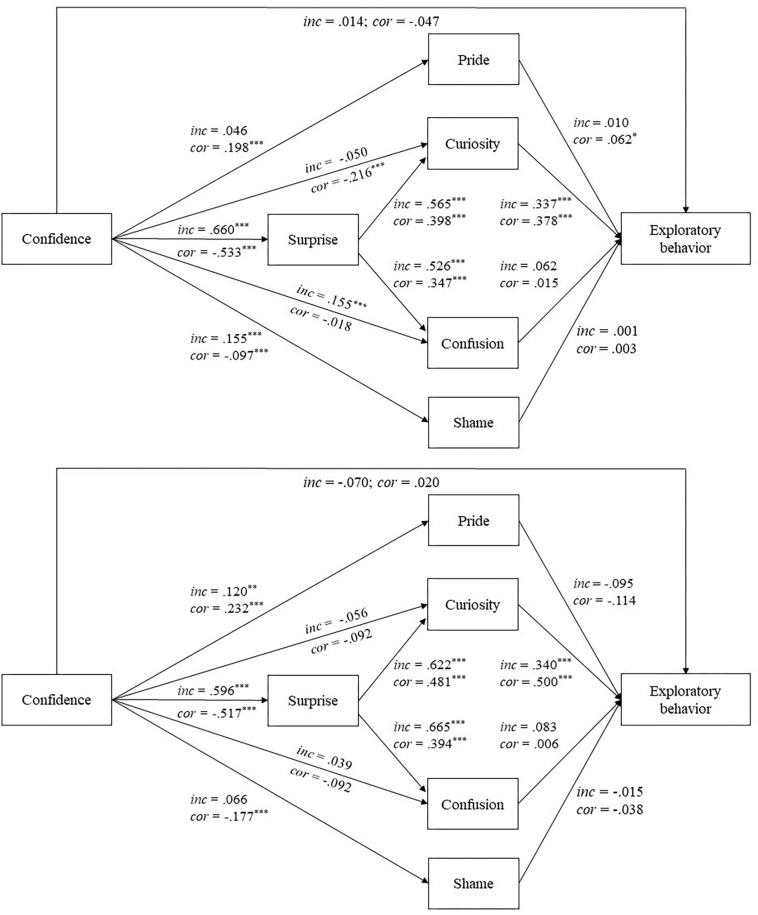
Results of Model 2 in Studies 1 **(upper panel)** and 2 **(lower panel)**. inc, path coefficients for incorrect answers (Model 2a); cor, path coefficients for correct answers (Model 2b). Residuals and correlations between emotions are not depicted. ^∗^*p* < 0.05, ^∗∗^*p* < 0.01, ^∗∗∗^*p* < 0.001.

Model 2a (confidence in incorrect answers) fitted well to the data: χ^2^(1) = 1.223, *p* = 0.269; RMSEA = 0.012; CFI = 1.00; SRMR_within_ = 0.003. Model 2b (confidence in correct answers) showed an equally good fit: χ^2^(1) = 1.148, *p* = 0.284; RMSEA = 0.009; CFI = 1.00; SRMR_within_ = 0.003; see Figure 2 and Supplementary Table S1. In line with Hypothesis 1, surprise was significantly positively predicted by high-confidence errors and significantly negatively predicted by confidence in correct answers. In both models surprise significantly positively predicted curiosity and confusion. Confidence in correct answers significantly positively predicted pride but significantly negatively predicted shame. In contrast, high-confidence errors significantly positively predicted shame but were not significantly related to pride.

In line with Hypothesis 2, curiosity significantly positively predicted exploratory behavior in both models. In contrast, confusion did not significantly predict exploratory behavior; however, in case of incorrect answers the path coefficient was positive and marginally significant (*p* = 0.060). Surprise had significant positive indirect effects on exploratory behavior that were mediated by curiosity but not by confusion in both models. However, the indirect effect mediated by confusion in case of incorrect answers was again marginally significant (*p* = 0.067). Overall, these findings provide partial support for the surprise-exploration relation hypothesis. In line with Hypothesis 5, pride positively predicted exploratory behavior after correct answers, whereas shame did not significantly relate to exploratory behavior.

In line with Hypothesis 3, high-confidence errors significantly positively predicted exploratory behavior. More specifically, we found significant indirect effects of confidence in incorrect answers on exploratory behavior that were mediated by surprise and curiosity but not by surprise and confusion. However, the confusion path was marginally significant (*p* = 0.066). Further supporting mediation, the direct effect of confidence in incorrect answers on exploratory behavior was not significant. In addition, there was a significantly indirect negative effect of confidence in correct answers on exploratory behavior mediated by surprise and curiosity but not by surprise and confusion. There was no significant direct effect of confidence in correct answers on exploratory behavior.

### Discussion

Study 1 aimed to exactly replicate Vogl et al.’s (2019, Study 3) findings. We found the same patterns of antecedents for epistemic emotions and achievement emotions as Vogl et al. (2019): High-confidence errors positively predicted epistemic emotions, whereas success and failure predicted achievement emotions (Pekrun, 2018). However, the intensity of pride and shame depended also on participants’ confidence in their answer: High-confidence errors produced more shame than low-confidence errors. Conversely, participants experienced more pride in correct answers accompanied by high than low confidence. However, the Accuracy × Confidence interaction predicted epistemic emotions more strongly than achievement emotions.

In line with hypotheses, surprise and curiosity positively predicted knowledge exploration in case of incorrect and correct answers. The effects of surprise were mediated by curiosity. The significant positive effect of confusion on exploration in case of incorrect answers reported by Vogl et al. (2019, Study 3) was replicated in terms of the direction of the effect, but not in terms of significance. The path coefficient (ß = 0.062) and the 95% confidence interval (95% CI = [−0.003; 0.127]) indicate a positive trend. Furthermore, the confidence interval largely overlaps with the corresponding confidence interval produced by the previous findings (Study 3 in Vogl et al., 2019: 95% CI = [0.026, 0.174]).

In addition, replicating Vogl et al.’s (2019) findings, high-confidence errors positively predicted exploration. As expected, curiosity was a mediator in the effect of errors on exploration. Conversely, exploratory behavior seems to be underminded if confidence in the accuracy of one’s answer is confirmed as evidenced by the negative effects of confidence in correct answers on exploratory behavior. It is important to note, however, that the results are correlational. Experimental evidence is needed to confirm the proposed temporal order of the emotions.

As expected, pride after correct answers promoted exploratory behavior. Conversely, pride did not predict exploration after incorrect answers incorrect answers because participants did not experience pride in this case (*M* = 1.11, *SD* = 0.35). In line with Vogl et al.’s (2019) findings, shame did not significantly predict exploration. These findings support the assumption that shame can have variable effects and does not have to be detrimental for knowledge generating behaviors.

In sum, the findings from Vogl et al. (2019, Study 3) were directly replicated by Study 1 of the present research. In the next step, however, the generalizability of the findings needs to be tested. Therefore, Study 2 was moved to a different setting.

## Study 2

Study 2 also aimed to directly replicate Study 3 from Vogl et al. (2019) by using the exact same target group of participants, materials, and procedure. However, we changed the study setting from online to lab to test the generalizability of the findings across these two settings. This study setting allowed also controlling for confounding effects and distracting stimuli.

### Method

#### Participants

The lab study was completed by 78 participants (59 women, *M*_age_ = 23.62, *SD* = 7.13) from a German university. As thanks for their participation, they could choose between credit as a subject or €10.

#### Materials, Procedure, and Measures

Study 2 used the same materials, procedure, and measures as Study 1. However, the study was conducted in a laboratory under controlled conditions. Ethical approval has been obtained by the Faculty of the first authors’ institution.

#### Data Analysis

We used multilevel structural equation modeling with Mplus 7.4 (Muthén and Muthén, 2017) to model descriptive statistics for within- and between-person relations between the study variables. However, to estimate Models 1 and 2, we needed to reduce the number of parameters given smaller sample size to avoid model convergence errors in this study. As such, we used the type = complex-option to estimate these models. Therefore, Level 2 relations were not explicitly modeled.

### Results

#### Preliminary Findings

Descriptive statistics and correlations are displayed in Table 1. Scores sufficient varied for all study variables. ICCs for emotions ranged from 0.157 to 0.323, indicating that within-person variation outweighed variation at the between-person level.

#### Origins of Epistemic and Achievement Emotions (Model 1)

Results for Model 1 are displayed in Tables 2, 3. The model was again saturated. Confirming high-confidence as antecedents epistemic emotions (Hypothesis 1) and replicating the Study 1 findings, accuracy and the Accuracy × Confidence interaction significantly negatively predicted surprise, curiosity, and confusion.

As expected, accuracy significantly positively predicted pride and significantly negatively predicted shame (Hypothesis 4). However, the Accuracy × Confidence interaction term also significantly positively predicted pride and significantly negatively predicted shame. These results indicate that participants were more proud in and less ashamed of correct answers they were confident in. However, the interaction term predicted epistemic emotions more strongly than achievement emotions (β range −0.324 to −0.527 for the epistemic emotions and 0.110 and −0.079 for pride and shame; see Tables 2, 3, respectively)

#### Outcomes and Relations of Epistemic and Achievement Emotions (Model 2)

Both models showed a good model fit with exception of the RMSEA: Model 2a (confidence in incorrect answers), χ^2^(1) = 8.360, *p* = 0.004; RMSEA = 0.095; CFI = 0.990; SRMR = 0.011; Model 2b (confidence in correct answers), χ^2^(1) = 34.206, *p* = 0.000; RMSEA = 0.212; CFI = 0.941; SRMR = 0.018 (see Figure 2 and Supplementary Table S1). As proposed in Hypothesis 1, high-confidence errors significantly positively predicted surprise, whereas confidence in correct answers significantly negatively predicted surprise. In both models surprise significantly positively predicted curiosity and confusion. In contrast to previous findings, pride was significantly positively predicted by confidence in correct as well as incorrect answers. As in Study 1, shame was significantly negatively predicted by confidence in correct answers.

As proposed in Hypothesis 2, curiosity significantly positively predicted exploration in both models, whereas confusion was not significantly related to exploratory behavior. The effect of surprise on exploratory behavior was significantly mediated by curiosity but not by confusion, after correct and incorrect answers. In contrast to Study 1 and Hypothesis 5, pride after correct answers did not significantly predict exploration. As expected, shame was not related to exploratory behavior.

In line with Hypothesis 3, high-confidence errors significantly positively predicted exploration. This indirect effect was significantly mediated by surprise and curiosity but not by surprise and confusion. The direct effects of confidence in incorrect and correct answers on exploration were not significant.

### Discussion

Goal of Study 2 was to directly replicate Vogl et al.’s (2019, Study 3) findings and to test their generalizability across settings. However, we need to keep in mind that the settings addressed in the present studies are a lab situation and a particular online environment. Additional research is needed to test the generalizability of the findings across other settings that might be more representative of daily life (such as knowledge exploration in books or libraries or an unrestricted online search). In line with previous findings and the results of Study 1, epistemic emotions were produced by high-confidence errors, whereas the achievement emotions pride and shame were predominantly triggered by success and failure, respectively (Pekrun, 2018). Nevertheless, the intensity of pride and shame was also influenced by the confidence in one’s reply. However, as in Study 1 and Vogl et al. (2019), the Accuracy × Confidence interaction predicted epistemic emotions more strongly than achievement emotions.

Replicating previous findings, surprise and curiosity positively promoted exploration, following incorrect and correct answers. Curiosity was a mediator in this effect. As in Study 1, the positive effect of confusion on exploration was replicated in terms of the direction of the effect but not in terms of significance. The path coefficient (ß = 0.083) and the overlap of the 95% confidence interval with the confidence intervals of the previous findings indicated once more a trend supporting a positive direct effect of confusion on knowledge generation (Study 1: 95% CI = [−0.003; 0.127]; Study 2: 95% CI = [−0.029; 0.195]; Study 3 in Vogl et al. (2019): 95% CI = [0.026, 0.174]). The non-significant result in case of correct answers is not surprising because confusion is not experienced in this case (*M* = 1.19, *SD* = 0.58).

Furthermore, exploratory behavior was elicited by high-confidence errors. As expected, the epistemic emotions surprise and curiosity (but not confusion) were mediators in this effect. Exploratory behavior was not related to confidence in correct answers. Nevertheless, experimental research is needed to confirm the temporal order of epistemic emotions.

In contrast to Study 1 and Vogl et al.’s (2019, Study 3) results, the positive effect of pride after correct answers on knowledge exploration could not replicated neither in terms of the direction of the effect (ß = −0.114, *p* = 0.077) nor in terms of significance. The 95% confidence interval of the path (95% CI = [−0.240; 0.012]) is not overlapping with the previous findings indicating a positive trend (Study 1: 95% CI = [0.011; 0.112]; Study 3 in Vogl et al. (2019): 95% CI = [0.027, 0.144]). Clearly, more research is needed to understand the effect of pride after correct answers on knowledge exploration. As expected, incorrect answers did not result in pride (*M* = 1.06, *SD* = 0.20). Consequently, pride did not predict exploratory behavior in this case either. Finally, as in Study 1 and findings by Vogl et al. (2019), exploratory behavior was not related to shame, supporting the assumption that shame does not necessarily have to be detrimental for knowledge generating behaviors.

## Meta-Analysis

Finally, we meta-analytically analyzed the estimates across the two present studies and Study 3 from Vogl et al. (2019) to get a more precise estimation of the effects and thereby a more accurate understanding of the origins, outcomes, and relations of the epistemic emotions surprise, curiosity, and confusion with the achievement emotions pride and shame.

### Method

The package metafor (Viechtbauer, 2010) produced for the R environment (version 3.2.2; R Core Team, 2015) was used to conduct the meta-analysis including the results of Vogl et al. (2019, Study 3) and both studies of this research. For each path of Models 1 and 2, we ran separate random-effects models to calculate the mean weighted effects based on the findings from the three studies.

### Results

#### Origins of Epistemic and Achievement Emotions (Model 1)

The mean weighted effect sizes ($\bar{ß}$) for the predictors of epistemic emotions and achievement emotions are displayed in Tables 4, 5, respectively. The meta-analytic findings confirmed that the Accuracy × Confidence interaction significantly negatively predicted epistemic emotions ($\bar{ß}$ range −0.337 to −0.515, Table 4). These findings support Hypothesis 1 claiming that epistemic emotions are triggered by high-confidence errors. In contrast, accuracy was the strongest predictor of achievement emotions (Hypothesis 4). Specifically, accuracy positively predicted pride ($\bar{ß}$ = 0.594, Table 5) and negatively predicted shame ($\bar{ß}$ = −0.375, Table 5). In addition, the meta-analytic findings showed that the Accuracy × Confidence interaction term also significantly predicted the experience of pride (positively) and shame (negatively). These findings indicate that the intensity of pride was also depended on one’s confidence in the answer. However, effect sizes for achievement emotions ($\bar{ß}$ = 0.119 and −0.126 for pride and shame, respectively) were much weaker than for epistemic emotions and confidence intervals of the effects of epistemic and achievement emotions did not overlap (see Tables 4, 5).

**TABLE 4:** Mean weighted effect sizes ($\bar{ß}$) for epistemic emotions in Model 1 across three studies.

	**Surprise**				**Curiosity**				**Confusion**			
			
**Predictor**	**$\bar{ß}$**	***p***	**95% CI**	***τ*^2^**	**$\bar{ß}$**	***p***	**95% CI**	***τ*^2^**	**$\bar{ß}$**	***p***	**95% CI**	***τ*^2^**
Accuracy	−0.448	<0.001	[−0.518; −0.377]	0.003	−0.424	<0.001	[−0.449; −0.398]	0.000	−0.467	<0.001	[−0.502; −0.433]	0.001
Confidence	0.142	<0.001	[0.094; 0.191]	0.002	−0.043	0.112	[−0.096; 0.010]	0.002	0.240	<0.001	[0.201; 0.280]	0.001
Accuracy × Confidence	−0.515	<0.001	[−0.536; −0.494]	0.000	−0.340	<0.001	[−0.381; −0.298]	0.001	−0.337	<0.001	[−0.357; −0.317]	0.000
Confidence in incorrect answers	0.651	<0.001	[0.621; 0.681]	0.000	0.365	<0.001	[0.315; 0.416]	0.001	0.508	<0.001	[0.436; 0.580]	0.003
Confidence in correct answers	−0.514	<0.001	[−0.550; −0.479]	0.000	−0.409	<0.001	[−0.446; −0.372]	0.000	−0.185	<0.001	[−0.236; −0.134]	0.001

**$\bar{ß}$*, mean-weighted standardized path coefficient; *p, p*-value; CI, confidence interval; *τ*^2^, between-study heterogeneity of effect sizes.*

**TABLE 5:** Mean weighted effect sizes ($\bar{ß}$) for achievement emotions in Model 1 across three studies.

	**Pride**				**Shame**			
		
**Predictor**	**$\bar{ß}$**	***p***	**95% CI**	***τ*^2^**	**$\bar{ß}$**	***p***	**95% CI**	***τ*^2^**
Accuracy	0.594	<0.001	[0.560; 0.629]	0.001	−0.375	<0.001	[−0.427; −0.323]	0.002
Confidence	0.102	<0.001	[0.080; 0.124]	0.000	0.088	<0.001	[0.050; 0.125]	0.001
Accuracy × Confidence	0.119	<0.001	[0.092; 0.146]	0.000	−0.126	<0.001	[−0.171; −0.081]	0.001
Confidence in incorrect answers	0.074	0.002	[0.028; 0.120]	0.001	0.169	0.002	[0.064; 0.274]	0.007
Confidence in correct answers	0.233	<0.001	[0.173; 0.294]	0.001	−0.090	0.022	[−0.168; −0.013]	0.004

**$\bar{ß}$*, mean-weighted standardized path coefficient; *p, p*-value; CI, confidence interval; *τ*^2^, between-study heterogeneity of effect sizes.*

#### Outcomes and Relations of Epistemic and Achievement Emotions (Model 2)

**FIGURE 3:**
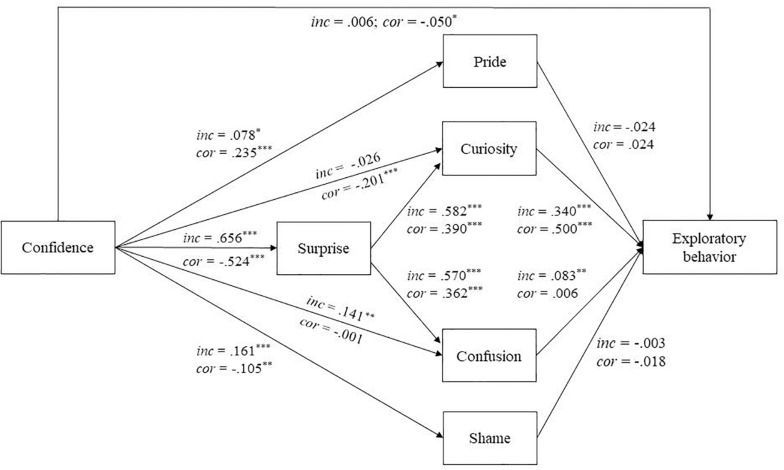
Mean weighted relations across three studies between confidence in answers, epistemic emotions, and exploration at the within-person level based on the meta-analytic findings. inc, path coefficients for incorrect answers (Model 2a); cor, path coefficients for correct answers (Model 2b). Residuals and correlations between emotions are not depicted. ^∗^*p* < 0.05, ^∗∗^*p* < 0.01, ^∗∗∗^*p* < 0.001.

Figure 3 and Supplementary Table S2 display the mean weighted effect sizes ($\bar{ß}$ for direct effects, $\bar{b}$ for indirect effects) for Model 2. Meta-analytic findings support Hypothesis 1 by confirming that high-confidence errors were a strong significant positive predictor of surprise ($\bar{ß}$ = 0.656). Conversely, surprise was significantly negatively predicted by confidence in correct answers. Curiosity and confusion were significantly positively predicted by surprise, after correct and incorrect answers. High-confidence errors significantly predicted pride but significantly negatively predicted shame. Vice versa, confidence in correct answers significantly positively predicted pride and significantly negatively predicted shame.

Furthermore, meta-analytic findings showed that curiosity and confusion significantly positively predicted exploratory behavior after incorrect answers (Hypothesis 2). Mean weighted effect sizes indicated a stronger effect of curiosity ($\bar{ß}$ = 0.330) than confusion ($\bar{ß}$ = 0.079). Curiosity also significantly predicted exploration after correct answers. In addition, exploratory behavior was significantly positively predicted by surprise. In more detail, the positive effects of surprise on exploration were mediated by curiosity and confusion, following incorrect answers. This mediation is additionally supported by the diminished direct effects of confidence in incorrect answers on curiosity ($\bar{ß}$ = −0.026) and confusion ($\bar{ß}$ = 0.141), respectively, as compared to Model 1. In case of correct answers, the significant positive indirect effect of surprise on exploration was mediated by curiosity only. Overall, these findings provide support for the surprise-exploration relation hypothesis (Hypothesis 2). In contrast to Hypothesis 5 and previous findings in the single studies, the meta-analytic findings did not support a positive effect of pride on exploratory behavior after correct answers. Exploratory behavior was not significantly predicted by shame either.

As expected, meta-analytic findings confirmed a significant positive effect of high-confidence errors on exploratory behavior mediated by surprise and curiosity as well as surprise and confusion (Hypothesis 3). In line with the direct effects, the mean weighted effect sizes indicated a stronger effect of surprise and curiosity ($\bar{ß}$ = 0.089) than surprise and confusion ($\bar{ß}$ = 0.020). The effect of confidence in correct answers on exploratory behavior was significantly mediated by surprise and curiosity only. In line with the mediation hypothesis, confidence in incorrect answers did not directly predict exploratory behavior. Confidence in correct answers significantly negatively predicted exploratory behavior.

### Discussion

The meta-analysis allowed a more precise estimation of the effects, revealed consistency of our findings, and helped to resolve inconsistencies regarding the effect of confusion across the present studies and Study 3 from Vogl et al. (2019). Future research drawing on different stimuli, different methods to induce epistemic emotions, or different types of exploratory or knowledge generating behavior, for instance, is needed to further evaluate the generalizability of our findings. Overall, the meta-analytic results support distinct patterns of antecedents for epistemic emotions and achievement emotions: High-confidence errors elicit epistemic emotions (Hypothesis 1), whereas pride and shame are triggered more strongly by success and failure, respectively (Hypothesis 4; Pekrun, 2018). Interestingly, the results confirm that the intensity of achievement emotions also depends on confidence in the answer: Pride was more intensely experienced if confidence in the correct answers was high and shame was more intensely experienced if confidence in the incorrect answer was high. However, epistemic emotions were more strongly influenced by the interaction of accuracy and confidence than achievement emotions [range of the mean weighted effect sizes ($\bar{ß}$ = −0.337 to −0.515 for the epistemic emotions and 0.119 and −0.126 for pride and shame); see Tables 4, 5, respectively].

In line with Hypothesis 2, surprise, curiosity, and confusion positively predicted knowledge exploration, after incorrect answers. Curiosity and confusion were mediators in the effect of surprise. As expected, exploratory behavior was positively predicted by high-confidence errors, while surprise and curiosity as well as surprise and confusion were mediators in this effect (Hypothesis 3). Conversely, exploratory behavior was reduced when participants’ expectations concerning the accuracy of their answer were confirmed.

With regard to the effect of pride after correct answers on exploration, the meta-analytic findings support Hypothesis 5 in terms of the direction of the effect, but not in terms of significance. However, the mean weighted effect size was very small ($\bar{ß}$ = 0.024). Finally, shame did not significantly predict exploratory behavior. These findings suggest that shame may not necessarily be negative for knowledge generating behaviors.

## General Discussion

Studies 1 and 2 largely replicated the findings by Vogl et al. (2019) and confirmed the robustness of their results and their generalizability across settings (online and lab), with the exception of the effects of confusion after incorrect answers and pride after correct answer on knowledge exploration. In addition, the meta-analysis allowed a more precise estimation of the effects. In sum, the results confirm distinct patterns of antecedents for epistemic and achievement emotions as well as positive effects of the epistemic emotions surprise, curiosity, and confusion on knowledge exploration.

### Origins of Epistemic and Achievement Emotions

Both new studies conducted in this research largely replicated the findings from Vogl et al. (2019, Study 3). In addition, the meta-analysis supported that epistemic emotions surprise, curiosity, and confusion were elicited by cognitive incongruity induced by high-confidence errors (Berlyne, 1954; Loewenstein, 1994; D’Mello and Graesser, 2012; Pekrun and Stephens, 2012; Silvia, 2013), whereas achievement emotions were more strongly related to success and failure in the trivia task (i.e., answering a statement correctly vs. incorrectly; mean weighted effect sizes ($\bar{ß}$) for pride and shame 0.594 and −0.375, respectively, Table 5; e.g., Pekrun et al., 2017a, b). Even though participants’ confidence in the answers also influenced the intensity of pride and shame, the impact was much stronger for epistemic emotions than for achievement emotions (range of the mean weighted effect sizes ($\bar{ß}$) for the Accuracy × Confidence interaction predicting epistemic emotions −0.337 to −0.515, Table 4, and pride and shame 0.119 and −0.126, respectively, Table 5). In sum, these results confirm cognitive incongruity as “prime driver of epistemic emotions” (Vogl et al., 2019, p. 13) and highlight the connection of metacognitive processes and epistemic emotions.

The results further support the within-person relations of epistemic emotions as suggested by Vogl et al. (2019): the findings support surprise as an antecedent of curiosity and confusion (Loewenstein, 1994; D’Mello and Graesser, 2012). However, the relations observed in the three studies relevant for this research are of correlational nature. Experimental research is needed to test the causal relationship of surprise, curiosity, and confusion in more detail.

### Outcomes of Epistemic and Achievement Emotions

The findings are in line with prior research on positive effects of curiosity and knowledge exploration (e.g., Litman et al., 2005) and support strong effects of curiosity on exploration after correct and incorrect answers. These effects were consistent across previous findings and both present studies. The results indicate that after incorrect answers cognitive incongruity triggers exploratory behavior mediated by surprise and curiosity. It is up for future research to investigate the origins of curiosity after correct answers. For example, personality traits may play an important role in the experience of state curiosity after correct and incorrect answers (e.g., epistemic beliefs or openness to experience; Muis et al., 2015a; Gocłowska et al., 2017; Fayn et al., 2019).

The findings for the effect of confusion on knowledge exploration after incorrect answers were less consistent across the three studies, including a significant positive effect in Study 3 from Vogl et al. (2019) and positive but non-significant effects in the present studies. However, confidence intervals of all three studies largely overlapped (Study 1: 95% CI = [−0.003; 0.127], Study 2: 95% CI = [−0.029;0.195], Study 3 from Vogl et al. (2019): 95% CI = [0.026, 0.174]). Finally, meta-analysis revealed a significant positive effect of confusion on knowledge exploration after incorrect answers ($\bar{ß}$ = 0.079). This supports the interpretation of Vogl et al. (2019) that the differences in the effect sizes of the different single studies may have been caused by sampling error. Nevertheless, it should be kept in mind that the positive effects of confusion were small and that the possibility to explore information might be essential for positive effects of confusion on knowledge generation. It is up for future research to explore the effects of confusion in situations, in which exploration is not possible (e.g., exams). Therefore, future research needs to investigate the impact of confusion on other types of knowledge generating behaviors to test the robustness and generalizability of the findings.

Regarding achievement emotions, results were mixed. Study 1 replicated a significant positive effect of pride after correct answers on knowledge exploration (Study 1: β = 0.062, 95% CI = [0.011; 0.112]; Study 3 from Vogl et al. (2019): β = 0.085, 95% CI = [0.027; 0.144]), whereas Study 2 revealed a non-significant negative relationship between these variables (Study 2: β = −0.114, 95% CI = [−0.240; 0.012]). Taken together, meta-analysis indicated that pride after correct answers may not relate to knowledge exploration (meta-analysis: $\bar{ß}$ = 0.024, 95% CI = [−0.084; 0.132]). Sampling error might explain the highly inconsistent findings across the three single studies. However, clearly more research is needed to investigate these contrary effects. The inclusion of more than three effect sizes in the meta-analysis will strengthen the overall power and with that the precision of the estimation of the effect. Nevertheless, it might be worthwhile investigating if the effects of pride differ in high-stakes achievement contexts, in which pride is usually studied (for an overview see Pekrun and Stephens, 2012), in contrast to low-stakes epistemic contexts as conceptualized in the present studies. In contrast to the findings for pride, we replicated the results for shame in both present studies. Meta-analytic findings additionally support the notion that shame did not impact knowledge exploration. This suggests in line theory and empirical evidence (Turner and Schallert, 2001; Pekrun and Stephens, 2012) that this negative emotion may not necessarily be detrimental for learning.

In sum, the findings confirm the robustness of the results from Vogl et al. (2019) and their generalizability across both settings (online and lab). They confirm high-confidence errors as predictors of epistemic emotions, and accuracy as predictor of achievement emotions. In addition, they support positive effects of all three epistemic emotions on the exploration of knowledge. In addition, the meta-analytic findings help to understand the effects of confusion on knowledge exploration and indicate a positive effect of this negative emotion on knowledge generation. However, the relationship between achievement emotions and epistemic behavior needs to be investigated in more detail.

### Implications for Research and Practice

Generally, empirical findings based on between-person data cannot be used to infer conclusions about the within-person relations proposed by emotion theories. While a between-person approach can be used to examine individual differences in the antecedents and effects of emotions, within-person analyses provide an understanding of the structures and variations of emotional states within individuals over time in response to different task characteristics (e.g., Tsai et al., 2008; Murayama et al., 2011). As such, only within-person analyses can provide evidence on the validity of emotion theories that predict within-person relations between variables. Nevertheless, between-person designs still dominate the existing body of research (see, e.g., Voelkle et al., 2014; Murayama et al., 2017). We used within-person analyses to investigate relations, origins, and outcomes of epistemic and achievement emotions. Therefore, the present results directly support emotion theories on epistemic and achievement emotions (e.g., Scherer, 2009; D’Mello and Graesser, 2012; Pekrun, 2018) that describe psychological mechanisms within-persons and highlight the robustness of these effects.

The present research directly replicated the findings from Vogl et al. (2019, Study 3) and provides meta-analytic estimates of antecedents and effects of epistemic emotions based on a synthesis of three independent studies. Such integrative evidence is pivotal as it provides more precise estimates based on increased statistical power, and can be systematically expanded using evidence from additional replication studies. Conceptual replications are needed to further probe the generalizability of our findings and bolster our underlying theoretical assumptions. Conceptual replications test the robustness of the finding across a range of variable conditions by intentionally and systematically altering the design of the original study (e.g., Schmidt, 2009). With regard to the present research, the following steps may be particularly important: First, the robustness of the findings needs to be tested across different sets of stimuli to make sure that the effects are not triggered by inherent characteristics of the materials used in the studies (Westfall et al., 2015). In addition, methods to induce cognitive incongruity should be systematically altered in future studies. Based on previous research, confronting participants with contradictory information (Muis et al., 2015a) or with breakdown scenarios (D’Mello and Graesser, 2014) may provide viable alternative induction approaches for testing if cognitive incongruity will reliably trigger epistemic emotions. Furthermore, with regard to probing the robustness of effects of epistemic emotions, different indicators of knowledge exploration should be examined. Herein, indicators reflecting exploratory behaviors that are more common in daily life can also strengthen conclusions about the ecological validity of the effects. For example, knowledge exploration could be measured by tracing participants’ searches for information in an online learning environment, or by observing learners’ physical exploration of additional information in books or by seeking help from others. To be able to generalize the findings even more broadly, the role of epistemic emotions during a single lesson, a seminar, or even the whole course of studies needs to be analyzed to understand their total impact on knowledge generation. The present findings also have important applications for practice. The results indicate positive effects of positive and negative epistemic emotions: Curiosity and confusion can promote knowledge exploration. In addition, they shed light on the origins of emotions. This knowledge is important for designing practical interventions in educational contexts (e.g., school and university). For example, they suggest that epistemic emotions could be triggered in the classroom by challenging naïve theories to promote engagement with the learning material. Beyond the educational context, violation of expectations could be used more broadly to trigger epistemic emotions and to encourage persons to engage with information (i.e., surprising news headlines).

## Data Availability Statement

The raw data supporting the conclusions of this manuscript will be made available by the authors, without undue reservation, to the reviewers upon request. The datasets for the present studies will be made publicly available in OSF upon acceptance of the manuscript (https://osf.io/dsx8f/).

## Ethics Statement

The studies involving human participants were reviewed and approved by Research Ethics Committee of the Faculty of Psychology and Education of the University of Munich. The patients/participants provided their written informed consent to participate in this study.

## Author Contributions

EV, RP, KM, KL, and SS contributed to the conception and design of the study and manuscript revision, and read and approved the submitted version. EV and RP performed the statistical analysis. EV wrote the first draft of the manuscript. RP, KM, KL, and SS gave feedback and rewrote sections of the manuscript.

## Conflict of Interest

The authors declare that the research was conducted in the absence of any commercial or financial relationships that could be construed as a potential conflict of interest.
