# Supplementary material for: Surprise, Curiosity, and Confusion Promote Knowledge Exploration: Evidence for Robust Effects of Epistemic Emotions
Source: Front Psychol. 2019 Nov 12;10:2474. doi: 10.3389/fpsyg.2019.02474 (PMC6861443; doi:10.3389/fpsyg.2019.02474)
Supplement: Supplementary file 1 [file Table_1.docx]

**Supplemental Materials for**

**Surprise, Curiosity, and Confusion Promote Knowledge Exploration: Evidence for Robust Effects of Epistemic Emotions**

This file includes:

Table S1: Path coefficients in Model 2 in Studies 1 and 2

Table S2: Mean Weighted Effect Sizes ($\bar{ß}$) in Model 2 across Three Studies

Table S1

*Path coefficients in Model 2 in Studies 1 and 2*

|  | Incorrect answers | | | |  | Correct answers | | | |
| --- | --- | --- | --- | --- | --- | --- | --- | --- | --- |
| Path | b | ß | *p* | 95% CI |  | b | ß | *p* | 95% CI |
|  | *Study 1* | | | | | | | | |
| Conf-Sur (a) | .611 | .660 | .000 | [.623; .696] |  | -.327 | -.533 | .000 | [-.583; -.483] |
| Conf-Cur | -.037 | -.050 | .123 | [-.113; .014] |  | -.160 | -.216 | .000 | [-.263; -.168] |
| Conf-Con | .125 | .155 | .000 | [.097; .212] |  | -.006 | -.018 | .563 | [-.080; .044] |
| Conf-Pri | .009 | .046 | .059 | [-.002; .093] |  | .110 | .198 | .000 | [.135; .262] |
| Conf-Sha | .081 | .155 | .000 | [.101; .209] |  | -.017 | -.097 | .000 | [-.140; -.054] |
| Conf-Expl | .008 | .014 | .676 | [-.052; .081] |  | -.028 | -.047 | .095 | [-.102; .008] |
| Sur-Cur (b) | .455 | .565 | .000 | [.493; .636] |  | .482 | .398 | .000 | [.343; .453] |
| Sur-Con (c) | .459 | .526 | .000 | [.464; .589] |  | .173 | .347 | .000 | [.264; .429] |
| Cur-Expl (d) | .264 | .337 | .000 | [.276; .398] |  | .304 | .378 | .000 | [.321; .436] |
| Con-Expl (e) | .045 | .062 | .060 | [-.003; .127] |  | .029 | .015 | .588 | [-.038; .068] |
| Pri-Expl (h) | .028 | .010 | .801 | [-.066; .085] |  | .067 | .062 | .017 | [.011; .112] |
| Sha-Expl (i) | .002 | .001 | .950 | [-.041; .044] |  | .011 | .003 | .898 | [-.047; .054] |
| Sur-Cur-Expl (b + d) | .073 | --- | .000 | [.054; .093] |  | -.048 | --- | .000 | [-.060; -.036] |
| Sur-Con-Expl (c + e) | .013 | --- | .067 | [-.001; .026] |  | -.002 | --- | .584 | [-.007; .004] |
| Conf-Sur-Cur-Expl  (a + b + d) | .120 | --- | .000 | [.089; .152] |  | .147 | --- | .000 | [.113; .181] |
| Conf-Sur-Con-Expl  (a + c + e) | .021 | --- | .066 | [-.001; .043] |  | .005 | --- | .582 | [-.013; .023] |
|  | *Study 2* | | | | | | | | |
| Conf-Sur (a) | .589 | .596 | .000 | [.517; .676] |  | -.365 | -.517 | .000 | [-.595; -.438] |
| Conf-Cur | -.047 | -.056 | .298 | [-.162; .050] |  | -.077 | -.092 | .083 | [-.195; .012] |
| Conf-Con | .038 | .039 | .423 | [-.057; .135] |  | -.007 | -.017 | .723 | [-.111; .077] |
| Conf-Pri | .028 | .120 | .000 | [.056 ; .184 ] |  | .211 | .232 | .000 | [.133; .330] |
| Conf-Sha | .047 | .066 | .148 | [-.023; .154] |  | -.032 | -.177 | .000 | [-.253; -.101] |
| Conf-Expl | -.050 | -.070 | .274 | [-.196; .056] |  | .016 | .020 | .742 | [-.101; .141] |
| Sur-Cur (b) | .533 | .622 | .000 | [.532; .713 ] |  | .574 | .481 | .000 | [.388 ; .574] |
| Sur-Con (c) | .646 | .665 | .000 | [.579; .751] |  | .220 | .394 | .000 | [.256 ; .532] |
| Cur-Expl (d) | .287 | .340 | .000 | [.226; .454] |  | .475 | .500 | .000 | [.383 ; .617] |
| Con-Expl (e) | .062 | .083 | .147 | [-.029; .195] |  | .013 | .006 | .877 | [-.075 ; .087] |
| Pri-Expl (h) | -.285 | -.095 | .131 | [-.218; .028] |  | -.100 | -.114 | .077 | [-.240 ; .012] |
| Sha-Expl (i) | -.015 | -.015 | .805 | [-.134; .104] |  | -.167 | -.038 | .483 | [-.142 ; .067] |
| Sur-Cur-Expl (b + d) | .153 | --- | .000 | [.097; .209 ] |  | .272 | --- | .000 | [.198 ; .347] |
| Sur-Con-Expl (c + e) | .040 | --- | .145 | [-.014; .093] |  | .003 | --- | .876 | [-.033; .039] |
| Conf-Sur-Cur-Expl  (a + b + d) | .090 | --- | .000 | [.058; .122 ] |  | -.099 | --- | .000 | [-.135 ; -.064] |
| Conf-Sur-Con-Expl  (a + c + e) | .023 | --- | .146 | [-.008 ; .055] |  | -.001 | --- | .877 | [-.014; .012] |

*Note.* Conf = confidence. Sur = surprise. Cur = curiosity. Con = confusion. Pri = pride. Sha = shame. Expl = exploration. Letters in parentheses display paths predicted by the main hypotheses (see Figure 1).
b = unstandardized path coefficient. ß = standardized path coefficient. CI = confidence interval.

Table S2

*Mean Weighted Effect Sizes (*$\bar{ß}$*) in Model 2 across Three Studies*

|  | Incorrect answers | | | |  | Correct answers | | | |
| --- | --- | --- | --- | --- | --- | --- | --- | --- | --- |
| Path | $\bar{b}$ / $\bar{ß}$ | *p* | 95% CI | *τ^2^* |  | $\bar{b}$ / $\bar{ß}$ | *p* | 95% CI | *τ^2^* |
| Conf-Sur (a) | .656 | <.001 | [.630; .682] | .000 |  | -.524 | <.001 | [-.556; -.492] | .000 |
| Conf-Cur | -.026 | .306 | [-.076; .024] | .000 |  | -.201 | <.001 | [-.295; -.107] | .006 |
| Conf-Con | .141 | .003 | [.050; .233] | .005 |  | -.001 | .979 | [-.044; .043] | .000 |
| Conf-Pri | .078 | .021 | [.012; .143] | .002 |  | .235 | <.001 | [.183; .288] | .001 |
| Conf-Sha | .161 | .001 | [.063; .260] | .007 |  | -.105 | .002 | [-.169; -.040] | .002 |
| Conf-Expl | .006 | .796 | [-.039; .051] | .000 |  | -.050 | .017 | [-.092; -.009] | .000 |
| Sur-Cur (b) | .582 | <.001 | [.536; .628] | .000 |  | .390 | <.001 | [.298; .481] | .005 |
| Sur-Con (c) | .570 | <.001 | [.486; .654] | .004 |  | .362 | <.001 | [.303; .422] | .000 |
| Cur-Expl (d) | .330 | <.001 | [.287; .372] | .000 |  | .399 | <.001 | [.349; .450] | .001 |
| Con-Expl (e) | .079 | .001 | [.034; .124] | .000 |  | .029 | .090 | [-.005; .062] | .000 |
| Pri-Expl (h) | -.024 | .314 | [-.072; .023] | .000 |  | .024 | .664 | [-.084; .132] | .008 |
| Sha-Expl (i) | -.003 | .875 | [-.037; .032] | .000 |  | -.018 | .404 | [-.061; .025] | .000 |
| Sur-Cur-Expl  (b + d) | .101 | <.001 | [.061; .141] | .001 |  | .173 | <.001 | [.084; .261] | .006 |
| Sur-Con-Expl  (c + e) | .020 | .006 | [.006; .035] | .000 |  | .010 | .116 | [-.002; .022] | .000 |
| Conf-Sur-Cur-Expl  (a + b + d) | .089 | <.001 | [.056; .122] | .001 |  | -.058 | .001 | [-.091; -.024] | .001 |
| Conf-Sur-Con-Expl  (a + c + e) | .020 | <.001 | [.010; .0310] | .000 |  | -.004 | .099 | [-.010; .0010 ] | .000 |

*Note.* Conf = confidence. Sur = surprise. Cur = curiosity. Con = confusion. Pri = pride. Sha = shame. Expl = exploration. Letters in parentheses denote paths predicted by the main hypotheses (see Figure 1). $\bar{b}$ = mean-weighted unstandardized path coefficient for indirect effects. $\bar{ß}$ = mean-weighted path coefficient for direct effects. CI = confidence interval. *τ^2^*=between-study heterogeneity of effect sizes.
